# Reply to Marini et al.: Insect spill-over is a double-edged sword in agriculture

**DOI:** 10.1073/pnas.2219197120

**Published:** 2022-12-27

**Authors:** Jay A. Rosenheim, Emma Cluff, Mia K. Lippey, Bodil N. Cass, Daniel Paredes, Soroush Parsa, Daniel S. Karp, Rebecca Chaplin-Kramer

**Affiliations:** ^a^Department of Entomology and Nematology, University of California Davis, Davis, CA 95616; ^b^Department of Plant Biology, Ecology and Earth Sciences, Universidad de Extremadura, Badajoz 06006, Spain; ^c^Regional Office for Latin America and the Caribbean, Food and Agriculture Organization of the United Nations, Vitacura Santiago 7630000, Chile; ^d^Department of Wildlife, Fish, and Conservation Biology, University of California Davis, Davis, CA 95616; ^e^Institute on the Environment, University of Minnesota, St. Paul, MN 55108

Agroecologists have long suggested that increasing the size of agricultural fields, one of the main features of agricultural industrialization, worsens problems with insect pests (1, 2). Recent research has examined the effects of mean field size across landscapes, suggesting highly context-dependent outcomes (3, 4). But, despite elegant theory describing insect responses to the size of a particular focal crop field (5, 6), there is a surprising deficit of empirical research addressing the question: should farmers plant smaller fields to improve pest control? Our study addressed this knowledge deficit. We found that smaller fields are sometimes associated with ameliorated pest impacts (the conventional expectation), but more often are associated with unchanged or worsened pest impacts (7).

As Marini et al. (8) note, our work examined the size of a single focal field and not the mean size of fields in the surrounding landscape. Generating knowledge about focal field size is important, as individual farmers can adjust the size of their fields, but often cannot implement changes across landscapes. Nevertheless, we agree that landscape context is important and that additional research is needed to explore landscape-scale mean field size, as well as many other factors that could modulate focal field size effects. We further concur with Marini et al. (8) that fine-grained landscapes may result in “improved landscape complementation and the facilitation of spill-over of organisms between crop and non-crop patches.” This does not necessarily imply improved pest control, however. Enhanced resource complementation and spill-over of organisms are double-edged swords: they may augment not only predators but also pests.

We respectfully disagree with Marini et al. (8) that our results failed to consider landscape-level factors sufficiently. Our statistical models controlled for landscape context by i) including key landscape-level covariates (natural habitat remnants; amount of the focal crop); ii) fitting spatial smoothers that corrected for regional differences in pest abundance; and iii) including fixed effects for ranch identity. Controlling for ranch identity isolates the effect of focal field size while holding constant features of the broader surrounding landscape. Furthermore, if any landscape effects leaked through our attempts at statistical control, we would expect them to make our conclusions more conservative. Smaller fields are most often found in landscapes with other small fields; thus, if small-field landscapes enhance pest control, it should only have made it more likely that we would observe lower pest densities in smaller focal fields. We did not observe that.

The sampling design proposed by Marini et al. (8) has clear efficiencies for parsing potential interactions of local and landscape field size. Maximizing efficiency is important when researchers must gather data with their own hands. However, the ecoinformatics methods that we used capitalize on farmer-generated data, decentralizing the labor-intensive task of data collection and yielding larger (ca 100×) data sets that are likely to include a broad array of landscape contexts (9). Such datasets could readily be analyzed to examine interactions of local- and landscape-scale factors. We agree with Marini et al. (8) that such work is worth pursuing.
